# Depression is differentially related to cognitive and biomarker outcomes among Mexican Americans

**DOI:** 10.3389/fpsyt.2022.901403

**Published:** 2022-08-23

**Authors:** Sid E. O’Bryant, Melissa Petersen, James Hall, Leigh A. Johnson

**Affiliations:** ^1^Institute for Translational Research, University of North Texas Health Science Center, Fort Worth, TX, United States; ^2^Department of Family Medicine, University of North Texas Health Science Center, Fort Worth, TX, United States; ^3^Department of Pharmacology and Neuroscience, University of North Texas Health Science Center, Fort Worth, TX, United States

**Keywords:** Mexican American, depression, biomarkers, cognition, Alzheimer’s disease

## Abstract

**Introduction:**

Despite tremendous advancements in the research of Alzheimer’s disease (AD), Mexican Americans, who reflect 65% of the US Hispanic community, remain severely underrepresented in research. Our data demonstrate that risk factors for, and biomarkers of, AD are different among Mexican Americans as compared with non-Hispanic whites. Here, we examined the impact of depressive symptoms on cognitive and AD-relevant biomarker outcomes among the Mexican Americans.

**Methods:**

Data were examined from 1,633 (852 Mexican Americans and 781 non-Hispanic whites) of the Health and Aging Brain Study–Health Disparities (HABS–HD). Depression was assessed using the Geriatric Depression Scale while cognition was measured using detailed neuropsychological testing. Plasma biomarkers of Aβ40, Aβ42, total tau, and NfL were examined in addition to MRI-based neurodegeneration. PET amyloid data were available in a subset of participants.

**Results:**

Depressive symptoms were significantly associated with cognitive testing results among both Mexican Americans and non-Hispanic whites. However, depression was only significantly associated with cognitive outcomes and plasma biomarkers among the Mexican American *APOE*ε4 non-carriers.

**Discussion:**

Depressive symptoms are more commonly endorsed by Mexican Americans and these symptoms are more strongly associated with cognitive and AD-biomarker outcomes among this ethnic group. However, depression scores were only related to AD outcomes among *APOE*ε4 non-carriers within the Mexican American group. These findings can aid in the development of a population-informed precision medicine for treating and preventing cognitive loss among the Mexican Americans.

## Introduction

Alzheimer’s disease (AD) is the most common neurodegenerative dementia that disproportionately impacts underserved communities [(1), Alzheimer’s disease facts and figures; (2)]. In fact, the US Hispanic population is projected to experience the largest increase in AD and AD-related dementias (ADRDs) over the next several decades (3). Despite the rapid growth in the US Hispanic population, and the projected increase in AD/ADRDs among this community, Hispanics (65% of which are Mexican American) remain severely underrepresented in AD clinical research (2, 4) and clinical trials (5).

In our prior work, we have shown that AD biomarkers and risk factors are different among Mexican Americans as compared to non-Hispanic whites (4, 6–9). We have shown that Mexican Americans develop cognitive impairment (4, 7, 10) and MRI-based neurodegeneration (9) at significantly younger ages. However, these disparities are in the context of lower rates of amyloid positivity (4) and lower frequency of *APOE*ε4 genotype (7, 10). In addition, plasma-based biomarkers of AD also are different among Mexican Americans (8). Therefore, it is important to study other factors that are related to AD risk among the Mexican Americans.

In our prior work, we found that depressive scores were elevated significantly among the Mexican Americans as compared with the non-Hispanic whites across cognitive diagnoses (10) and those depressive symptoms were a stronger predictor of neuropsychological functioning among Mexican Americans (11). Given the substantial literature documenting the link between depression and risk for AD (12–20), this may be a risk factor that disproportionally impacts Mexican Americans. Therefore, in this study, we examined the link between depressive symptoms and neuropsychological as well as biomarker (MRI, PET, and plasma) outcomes among Mexican Americans as compared with non-Hispanic whites in the ongoing.

## Materials and methods

### Participants and assessment

The Health and Aging Brain Study–Health Disparities (HABS–HD; formally the Health and Aging Brain study among Latino Elders, HABLE study) study is an ongoing, longitudinal, community-based project examining health disparities in MCI and AD among Mexican Americans as compared with the non-Hispanic whites with recent expansion currently enrolling African Americans. HABS–HD methods have been published elsewhere (4) and are briefly outlined later. The data included in this study encompass Mexican American and non-Hispanic white participants since the recruitment of the African American participants is ongoing. Inclusion criteria for the study includes (1) self-reported ethnicity of African American, Mexican American, or non-Hispanic white, (2) willingness to provide blood samples, (3) capable of undergoing neuroimaging studies, (4) age 50 years and older, and (5) fluent in English or Spanish. Exclusion criteria include (1) Type 1 diabetes, (2) presence of active infection, (3) current/recent (12 months) cancer (other than skin cancer), (4) current severe mental illness that could impact cognition (other than depression), (5) recent (12 months) traumatic brain injury with loss of consciousness, (6) current/recent alcohol/substance abuse, (7) active severe medical condition that could impact cognition (e.g., end-stage renal failure, chronic heart failure, chronic obstructive pulmonary disease) and (8) current diagnosis of dementia other than AD. Participant recruitment for HABS–HD includes a community-based participatory research (CBPR) approach (21). The CBPR approach has been used successful as a recruitment modality for reaching underserved and minority populations. It involves collaborating with local communities through outreach (holding community events and seminars), word of mouth, marketing modalities (newspaper, television, and radio), and providing back information (clinical lab work, MRI clinical reads, and neuropsychological test results) to the participants and their healthcare providers. All the aspects of the study protocol can be conducted in Spanish or English. The HABS–HD study is conducted under IRB-approved protocols and each participant (or his/her legal representative) signs written informed consent. All the HABS–HD data are available to the scientific community through the UNTHSC Institute for Translational Research (22).^1^

### Clinical

An interview is conducted as part of the HABS–HD protocol which includes an interview and neuropsychological testing with the following battery: Mini-mental state examination (MMSE) (23), Wechsler Memory Scale, 3rd Edition (WMS-III) Digit Span and Logical Memory (24), Digit Symbol Substitution, Trail Making Test Parts A and B (25), Spanish–English Verbal Learning Test (SEVLT) (26), Animal Naming (semantic fluency) (26), FAS (phonemic fluency) (26), the American National Adult Reading Test (English speakers) (27), and Word Accentuation Test (Spanish speakers) (28). An informant interview was also conducted for completion of the Clinical Dementia Rating (CDR) acale (29) by the clinicians with expertise in dementia to evaluate for functional declines. Depressive symptoms were assessed using the 30-item Geriatric Depression Scale (30).

### Blood biomarkers

Blood samples were collected, processed, and stored per previously published international guidelines (31). Assay preparation was completed using a custom-automated Star Plus system from Hamilton Robotics. Plasma markers of amyloid (Aβ_40_, Aβ_42_), tau (total-tau) and neurodegeneration [neurofilament light (NfL)] were assayed using the ultra-sensitive Simoa (single molecule array technology platform XD-X (Quanterix.com) (4, 8, 9). APOE genotyping was performed using commercially available TaqMan Genotyping Kits for rs429158 and rs7412 using TaqMan GTXpress Master Mix (Thermo Fisher Scientific). Target amplification and detection were performed using the 7500 Real-Time PCR System (Applied Biosystems). Genotypes were called according to combined allele amplification results at the two SNPs as follows (rs429358, rs7412): ε2/ε2- T,T; ε2/ε3- T,CT; ε2/ε4- CT,CT; ε3/ε3- T/C; ε3/ε4- CT,C; ε4/ε4- C,C. Positive controls (individuals of known, independently typed APOE genotypes) and negative controls were included on all runs. APOE genotypes frequencies were confirmed to be in the Hardy–Weinberg equilibrium.

### Neuroimaging

Magnetic resonance imaging. The HABLE MRI protocol is based on that of ADNI3 using a 3T Siemens Magnetom SKYRA whole-body scanner. We acquired the following scan sequences: T1-weighted whole brain volumetric spoiled magnetization-prepared rapid gradient (MPRAGE), whole brain volumetric fluid-attenuated inversion recovery (FLAIR), susceptibility-weighted imaging (SWI), diffusion tensor MRI (dMRI), 3D arterial spin labeling (3DPASL), resting-state functional (rsfMRI), and high resolution (0.4 × 0.4 mm × 2 mm) T2-weighted hippocampal high resolution (HHR) scans. For this study, the neurodegeneration (i.e., N) component of the AT(N) framework (32) was examined as outlined by Jack et al. (32) as the “meta-ROI,” which comprises the surface-area weighted average of the mean cortical thickness in individual ROIs of the entorhinal cortex, fusiform, inferior temporal gyri, and middle temporal gyri. N^+^ was determined based on a cut-off of 2.68 mm for cortical thickness (32). Participants who failed quality checks [quality assurance (QA)] for the FreeSurfer software version 5.3.0 segmentation for at least one of the individual ROI sections (referenced earlier) were excluded when calculating meta-ROI. Meta-ROI was calculated based on the sum of each region in each hemisphere * the surface area for that region divided by the sum of surface areas for all regions included.

*Positron emission tomography amyloid (Neuraceq; aka florbetaben)*. PET amyloid was available on a subset of 34 Mexican Americans and 22 non-Hispanic whites. PET amyloid was conducted using Neuraceq with Siemens Biograph Vision 450 whole-body PET/CT scanner following the ADNI3 protocols. In brief, participants are injected with an 8.1 mCi (± 10%) bolus of Neuraceq. A 4-frame by 5-min (20 min total) dynamic emission acquisition is started 90 min post injection following the acquisition of a low-dose CT scan used for attenuation correction. The emission images are processed by iterative reconstruction, 4 iterations, and 16 subsets. FreeSurfer-defined regions (frontal, anterior/posterior cingulate, lateral parietal, and lateral temporal cortex) were used to derive a summary cortical ROI. Normalization to the whole cerebellum reference region was conducted to obtain global standardized update value ratios (SUVR). An SUVR of 1.08 was used to define positivity.

### Diagnostic classification

Cognitive diagnoses were assigned algorithmically (decision tree) and verified at consensus review as follows: *Normal control (NC)* = no cognitive complaints, CDR sum of boxes score of 0 (33, 34), and cognitive tests scores broadly within normal limits [i.e., performance greater than that defined as meeting diagnostic criteria for MCI (i.e., =1.5 SDs below the normative range)]; *Mild cognitive impairment (MCI)*: cognitive complaint (self or other), CDR sum of boxes score between 0.5 and 2.0 (33, 34) and at least one cognitive test score falling = 1.5 SDs below normative ranges; *Dementia*: CDR sum of boxes score =2.5 (33, 34) and at least two cognitive test scores 2 SDs below normative ranges.

### Statistical analyses

Statistical analyses were conducted in SPSS 25 (IBM). Chi-square and ANOVA were utilized to compare groups on demographic variables. Linear regression models were run to examine the link between GDS scores and cognitive and biomarker outcomes with age, gender, education, and cognitive diagnosis (i.e., control, mild cognitive impairment, dementia) entered as covariates. Logistic regression models were run to determine the impact of each depression on the risk for diagnosis of MCI and dementia. Analyses were run for the entire cohort and then split by ethnicity and finally by ethnicity × *APOE*ε4 genotype (except for amyloid PET results). Statistical significance was set at *p* < 0.05.

## Results

### Descriptive characterization

As of November 2021, a total of 1,633 participants were enrolled with all required data to be included in the current analyses (852 MAs and 781 NHWs). The Mexican American group was significantly younger (*p* < 0.001) and obtained fewer years of formal education (*p* < 0.001) than non-Hispanic whites. There was also a significant gender difference between groups with a higher number of females included among those who self-reported as Mexican American (*p* < 0.001). Regarding neuropsychological test performance, mean differences were found between ethnic groups with Mexican Americans performing lower across all cognitive domains (*p* < 0.001). The Mexican American group endorsed significantly higher depressive symptoms as compared to non-Hispanic whites (*p* < 0.001) (see Table 1).

**TABLE 1:** Descriptive statistics of c?ohort.

	Total cohort *N* = 1,614	Mexican American *N* = 853	Non-Hispanic white *N* = 781
Age, mean (*SD*)Range	66.47 (8.76)50 – 92	63.83 (7.98)50–91	69.35 (8.65)**50–92
Education, mean (*SD*)Range	12.37 (4.81)0–20	9.51 (4.61)0–20	15.50 (2.55) **0–20
Gender, % female	61%	66%	54%**
GDS-30 item	5.68 (5.87)	6.58 (6.32)	4.69 (5.18)**
WMS-III Digit Span, mean (*SD*)Range	13.69 (4.31)0–29	11.43 (1.97)0–25	16.15 (3.67) **6–29
Trail making test part A, mean (*SD*)Range	43.96 (25.39)15.00–150.00	50.79 (29.22)16.00–150.00	36.53 (17.65) **15.00–150.00
Trail making test part B, mean (*SD*)Range	128.59 (85.43)25.00–300.00	161.17 (93.45)25.00–300.00	93.77 (58.53) **25.00–300.00
FAS, mean (*SD*)Range	31.84 (12.25)0–68	27.10 (10.99)0–65	37.00 (11.45) **2–68
Animals, mean (*SD*)Range	17.48 (5.17)0–37	16.27 (4.79)0–33	18.81 (5.24) **0–37
WMS-III logical memory I, mean (*SD*)Range	35.23 (12.02)0–69	30.74 (10.65)0–58	40.11 (11.52) **0–69
WMS-III logical memory II, mean (*SD*)Range	21.29 (8.99)0–44	18.48 (8.10)0–41	24.33 (8.92) **0–44
SEVLT 1-5 total, mean (*SD*)Range	30.74 (9.08)0–53	28.91 (8.32)0–53	32.73 (9.44) **3–53
SEVLT delayed recall, mean (*SD*)Range	7.61 (3.46)0–15	6.96 (3.30)0–15	8.30 (3.48) **0–15

WMS, Wechsler Memory Scale; SEVLT, Spanish English Verbal Learning Test.

**p < 0.001.

### Neuropsychological outcomes

In the total cohort, GDS scores were associated with the poorer performance in language (FAS, *t* = -2.32, *p* = 0.02), processing speed (SDMT, *t* = -3.64, *p* < 0.001), and immediate memory (SEVLT 1-5, *t* = -1.21, *p* = 0.004). However, among Mexican Americans, GDS scores were significantly associated with poorer scores in processing speed (Trails A *t* = 2.46, *p* = 0.01, SDMT *t* = -3.26, *p* = 0.001), executive functioning (Trails B, *t* = 2.40, *p* = 0.02), language (FAS, *t* = -3.54, *p* < 0.001), immediate memory (SEVLT 1-5 *t* = -3.12, *p* = 0.002), and delayed memory (SEVLT Delayed *t* = -2.28, *p* = 0.02). Among non-Hispanic whites, GDS scores were only associated with poorer processing speed (SDMT *t* = -2.61, *p* = 0.009).

When split by *APOE*ε4 genotype, results changed. In fact, GDS scores were only significantly associated with neuropsychological test performance among Mexican Americans who were *APOE*ε4 negative. Among *APOE*ε4 non-carrier Mexican Americans, GDS scores were associated with poorer performance in the areas of processing speed (Trails A *t* = 2.73, *p* = 0.007, SDMT *t* = -3.47, *p* < 0.001), executive functioning (Trails B *t* = 2.09, *p* = 0.04), language (FAS *t* = -3.82, *p* < 0.001) as well as immediate (WMS-III LM1 *t* = -2.07, *p* = 0.04; SEVLT 1-5 *t* = -4.03, *p* < 0.001) and delayed (SEVLT Delayed *t* = -2.84, *p* = 0.005 with WMS-III LM2 *t* = -1.76, *p* = 0.08 approaching significance). Among non-Hispanic whites, GDS scores were associated with significantly worse scores in the language (FAS *t* = -2.04, *p* = 0.04) among *APOE*ε4 carriers and poorer immediate memory (SEVLT 1-5 *t* = -2.05, *p* = 0.04) among *APOE*ε4 non-carriers.

### Imaging biomarkers

The GDS scores were not associated with neurodegeneration (*via* MetaROI) among either ethnic group, including if split by *APOE*ε4. Among Mexican Americans, GDS scores were significantly associated with the frontal amyloid burden (*t* = -2.01, *p* = 0.04) with a trend toward significance among anterior/posterior cingulate (*t* = -2.02, *p* = 0.05) and global amyloid burden (*t* = -1.83, *p* = 0.08). GDS scores were not associated with cerebral amyloid burden among non-Hispanic whites.

### Plasma biomarkers

In the total cohort, GDS scores were associated with plasma Aβ_40_ (*t* = 3.24, *p* = 0.001) and Aβ_42_ (*t* = 3.52, *p* < 0.001) levels. Among Mexican Americans, GDS scores were significantly associated with both plasma Aβ_40_ (*t* = 2.09, *p* = 0.04) and Aβ_42_ (*t* = 3.62, *p* < 0.001) levels; however, GDS scores were only significantly associated with Aβ_40_ levels (*t* = 2.39, *p* = 0.02) among non-Hispanic whites. When split by *APOE*ε4 genotype, GDS scores were only associated with plasma Aβ_42_ levels among Mexican American *APOE*ε4 non-carriers (*t* = 3.49, *p* < 0.001). However, among non-Hispanic whites, GDS Scores were significantly associated with plasma Aβ_40_ levels (*t* = 2.44, *p* = 0.02) among *APOE*ε4 non-carriers but plasma total tau (*t* = 2.64, *p* = 0.009) levels among *APOE*ε4 carriers.

### Risk for cognitive impairment

The GDS scores were associated with increased risk for diagnosis of MCI (OR = 1.06, 95% CI 1.04–1.09, *p* < 0.001) and dementia (OR = 1.09, 95% CI 1.06–1.12, *p* < 0.001) in the full cohort. Among Mexican Americans, GDS scores were associated with a significantly increased risk for diagnosis of MCI (OR = 1.05, 95% CI 1.02–1.08, *p* < 0.001) and dementia (OR = 1.09, 95% CI 1.05–1.13, *p* < 0.001). Among non-Hispanic whites, GDS scores were associated with a significantly increased risk for diagnosis of MCI (OR = 1.09, 95% CI 1.05–1.14, *p* < 0.001) and dementia (OR = 1.09, 95% CI 1.04–1.15, *p* < 0.001). However, the risk for MCI (OR = 1.05, 95% CI 1.02–1.08, *p* = 0.001) and dementia (OR = 1.11, 95% CI 1.06–1.16, *p* < 0.001) was only significant for Mexican American *APOE*ε4 non-carriers. On the other hand, among non-Hispanic white *APOE*ε4 carriers, GDS scores were associated with increased risk for diagnosis of MCI (OR = 1.11, 95% CI 1.03–1.20, *p* = 0.009) and dementia (OR = 1.11, 95% CI 1.04–1.19, *p* = 0.003), but GDS scores were only associated with MCI risk among *APOE*ε4 non-carriers (OR = 1.09, 95% CI 1.04–1.15, *p* < 0.001).

## Discussion

The current findings add to the extant literature documenting the depressive symptoms are more strongly related to AD-related outcomes among Mexican Americans as compared to non-Hispanic whites. In addition, the link between depressive symptoms and AD-related outcomes appears to be primarily among Mexican Americans who are *APOE*ε4 non-carriers.

In our prior work, we have demonstrated that the *APOE*ε4 genotype is less common among Mexican Americans across cognitive diagnostic categories as compared to non-Hispanic whites. Here, we extend upon that work by demonstrating that the link between depression and cognitive outcomes is only present among *APOE*ε4 non-carriers. In addition, the link between depression and plasma AD-biomarkers was also restricted to *APOE*ε4 non-carriers. The current sample size of participants with amyloid PET scans was too small to stratify by *APOE*ε4 status; however, the HABS-HD study is currently capturing amyloid (and tau) PET scans on the entire cohort and future work will examine this question.

Our team has conducted a series of studies examining the impact of depression on cognitive outcomes among Mexican Americans (14, 15, 35–37). In a recent study, Hall et al. demonstrated a significant link between plasma tau and depressive symptoms among 538 community-dwelling Mexican Americans (38). Johnson et al. have found that comorbid depression—diabetes (14), as well as depression + elevated inflammation (36), were associated with increased risk for cognitive impairment among Mexican Americans. Johnson et al. also found that depressive symptoms were associated with an increased risk for cognitive decline among community-dwelling Mexican Americans (37). The current work expands significantly on that prior work by demonstrating that the link between depression and cognition is only present among *APOE*ε4 non-carriers among Mexican Americans. In addition, the link between depression and AD-relevant biomarkers was also only among Mexican American *APOE*ε4 non-carriers.

In light of our prior work demonstrating that (1) *APOE*ε4 is less common among Mexican Americans (7, 39), (2) amyloid positivity rates are lower among Mexican Americans clinically diagnosed MCI and dementia (4), (3) Mexican Americans develop cognitive loss (4, 7, 39) and MRI-defined neurodegeneration (9) at younger ages, (4) Mexican Americans express higher rates of depressive symptoms (7, 10), it is possible that Mexican Americans are experiencing higher rates of non-AD dementia syndromes as defined by the AT(N) framework. In fact, our work suggests that there may be a significant subset of Mexican Americans who are suffering cognitive loss because of the depressive symptoms. If this subgroup of individuals can be identified, this can lead to a precision medicine approach to treating and preventing cognitive loss among this underserved community. Additional work is ongoing in the HABS–HD cohort to identify such a subgroup for comprehensive characterization for the generation of a novel clinical trial. In fact, in a small-scale pilot clinical trial (NCT02590874) among 19 Mexican Americans diagnosed with MCI and depression, anti-depressant intervention improved both cognitive and biomarker outcomes (manuscript in preparation).

There are weaknesses to the current study. First, the current data is cross-sectional in nature; however, HABS–HD is longitudinal with visit two assessments underway currently. Therefore, longitudinal analyses of the impact of depression on cognition will be the focus of future work. Second, the sample size of amyloid PET scans was too small to stratify by *APOE*ε4 genotype; however, the HABS–HD study is currently capturing longitudinal amyloid (and tau) PET scans. Therefore, future work will seek to examine the link between depressive symptoms and amyloid burden both cross-sectionally and longitudinally. A third weakness is the lack of inclusion of the African American participants. Combined, Mexican Americans, African Americans, and non-Hispanic whites make up approximately 75% of the US population and, therefore, examining these topics simultaneously across the three largest racial/ethnic groups would provide substantial information to the field. HABS–HD is currently enrolling 1000 African Americans and, therefore, future work will be conducted across all three racial/ethnic groups. In spite of these limitations, the current work adds substantially to the extant literature. Additional work is ongoing within the HABS–HD study to determine if and how the current work can be transitioned into a population-informed precision medicine model for treating and/or preventing cognitive loss among Mexican Americans.

## Data availability statement

The datasets presented in this study can be found in online repositories. The names of the repository/repositories and accession number(s) can be found below: UNTHSC Institute for Translational Research (ITR) website.

## Ethics statement

This study protocol was reviewed and approved by the UNTHSC IRB protocols UNTHSC 2016-128 and 2020-125. Each participant (or his/her legal representative) signed written informed consent to participate in the study.

## Author contributions

SO’B and LJ: conceptualization and design of the study, acquisition, analysis, and interpretation of data, drafting and revising the manuscript, final approval of the version to be published, agreement to be accountable for the accuracy and integrity of the work. JH and MP: design of the study, acquisition and interpretation of data, drafting and revising the manuscript, final approval of the version to be published, agreement to be accountable for the accuracy and integrity of the work. All authors contributed to the article and approved the submitted version.

## Footnotes

1
https://apps.unthsc.edu/itr/
